# Characterization of infections and hypogammaglobulinemia treated with the combination of pertuzumab and trastuzumab

**DOI:** 10.1007/s00280-019-03970-8

**Published:** 2019-10-22

**Authors:** Joanne E. Mortimer, Laura Kruper, Jae Jung, Louise Wong, Jill Cooper, Daphne Stewart, Samuel Chung, Kim Wai Yu, Sanjeet Dadwal, Yuan Yuan

**Affiliations:** 1 Department of Medical Oncology and Experimental Therapeutics, City of Hope Comprehensive Cancer Center, 1500 East Duarte Rd, Duarte, CA 91010 USA; 2 Department of Surgery, City of Hope Comprehensive Cancer Center, 1500 East Duarte Rd, Duarte, CA 91010 USA; 3 Department of Dermatology, City of Hope Comprehensive Cancer Center, 1500 East Duarte Rd, Duarte, CA 91010 USA; 4 Department of Pharmacy, City of Hope Comprehensive Cancer Center, 1500 East Duarte Rd, Duarte, CA 91010 USA; 5 Department of Infection Disease, City of Hope Comprehensive Cancer Center, 1500 East Duarte Rd, Duarte, CA 91010 USA

**Keywords:** Infections, Hypogammaglobulinemia, Pertuzumab, Trastuzumab

## Abstract

**Purpose:**

We update a patient series that reported a high incidence of infection with Gram-positive cocci in women treated with the combination of pertuzumab and trastuzumab and further characterize this clinical problem.

**Patients:**

Treating physicians and advanced practice partners identified women who developed infections while on treatment with pertuzumab and trastuzumab alone or in combination with chemotherapy and enrolled them onto this registry trial.

**Results:**

Between March, 2014 and May, 2017, 48 patients with HER2-positive breast cancers were reported to have 59 individual infections. The median age was 48 years. Twenty-four patients received neoadjuvant therapy, 17 were treated for metastatic disease, and 7 were treated in the adjuvant setting. Pertuzumab and trastuzumab were combined with carboplatin and docetaxel in 24 (49%) patients, docetaxel in 10 (21%), nab-paclitaxel in 12 (24%), and without other agents in 2 (4%). Granulocyte growth factors were administered in 24 (49%) patients and no patients were documented to be neutropenic. Folliculitis developed in 25 (52%) patients and was counted as a single infection. Abscesses developed at a number of sites in 24 (49%) patients, including a septic knee requiring total knee replacement. Paronychia occurred in 7 (15%) patients, and 5 (10%) developed cellulitis. When cultures were obtained, Gram-positive cocci were consistently identified. Hypogammaglobulinemia was documented in 14 (36%) of the 33 patients tested.

**Conclusions:**

Our data continue to support an increased risk of infections with Gram-positive cocci as a potentially serious adverse event in women treated with pertuzumab and trastuzumab.

## Introduction

We previously described the development of unique infections involving the skin and nails that we attributed to the addition of pertuzumab to trastuzumab-based chemotherapy in women with HER2-positive breast cancer. This problem often developed in association with hypogammaglobulinemia [1]. This report updates and expands our initial series, providing longer follow-up and further characterization of the problem.

## Methods

From March, 2014 until May, 2017, women who developed infections while on chemotherapy regimens that included both pertuzumab and trastuzumab were enrolled onto a registry trial approved by the Institutional Review Board (IRB). Patients were identified by the treating physician or advanced practice partner. Following our initial report, the care team had a heightened awareness of the potential for infectious complications and subsequent patients were generally identified and reported prospectively. The patients in this manuscript include the 18 women previously reported [1].

The clinical workup of the patients was not defined. Because we reported hypogammaglobulinemia in many of the initial patients, quantitative immunoglobulins were frequently checked at the time of the infection. Antimicrobial treatment was determined by the treating clinicians on a case-by-case basis, and the departments of infectious disease and/or dermatology were often consulted for treatment recommendations.

An episode of infection was defined as an infection that prompted attention to the treating oncologist or advanced practice partner. Folliculitis was the most common, frequently involved multiple sites and it often recurred with subsequent cycles of treatment. We counted folliculitis as a single infection, even if the patient developed additional eruptions with subsequent cycles of treatment. In instances where the attribution of the infection to systemic therapy was in question, the infectious disease consultant (SD) provided the attribution.

## Results

### Characteristics and demographics

Forty-eight patients developed 59 individual episodes of infection. Their characteristics are summarized in Table 1. The median age was 48 years. Twenty-four (50%) patients received pertuzumab/trastuzumab-based chemotherapy in the neoadjuvant setting, 17 (35%) were treated for metastatic disease, and 7 (15%) were treated in the adjuvant setting. Two patients developed infections while on trastuzumab and pertuzumab alone, and 46 received treatment that included a taxane with 25 also receiving carboplatin. The majority of patients received granulocyte colony-stimulating factor (GCSF) agents. None of the patients were neutropenic when they presented with infection.

**Table 1:** Patient demographics

Number of patients	48
Age	48 years (25–73)
Setting	
Metastatic disease	17
Neoadjuvant	24
Adjuvant	7
Regimen	
PTH	10
PTCH	24
nabPT	12
PH	2
Type of infection (often multiple sites)	
Abscess	24 patients
Breast	1
Skin	1
Axilla	1
Port	2
Dental	1
Vaginal	1
Breast wound/seroma	2
Chest wall	1
Thigh	1
Thumb	1
Buttocks	4
Shoulder	1
Septic arthritis	1*
Paronychia	7
Folliculitis	25 patients
Scalp	13
Face	9
Abdomen	2
Arm	1
Paronychia	7
Cellulitis	5

*PTCH* pertuzumab, trastuzumab, carboplatin, and docetaxel, *PTH* pertuzumab, trastuzumab, and docetaxel, *nabPT* nab-paclitaxel, pertuzumab, and trastuzumab, *PH* pertuzumab and trastuzumab

*Patient required a total knee replacement

### Folliculitis

**Fig. 1:**
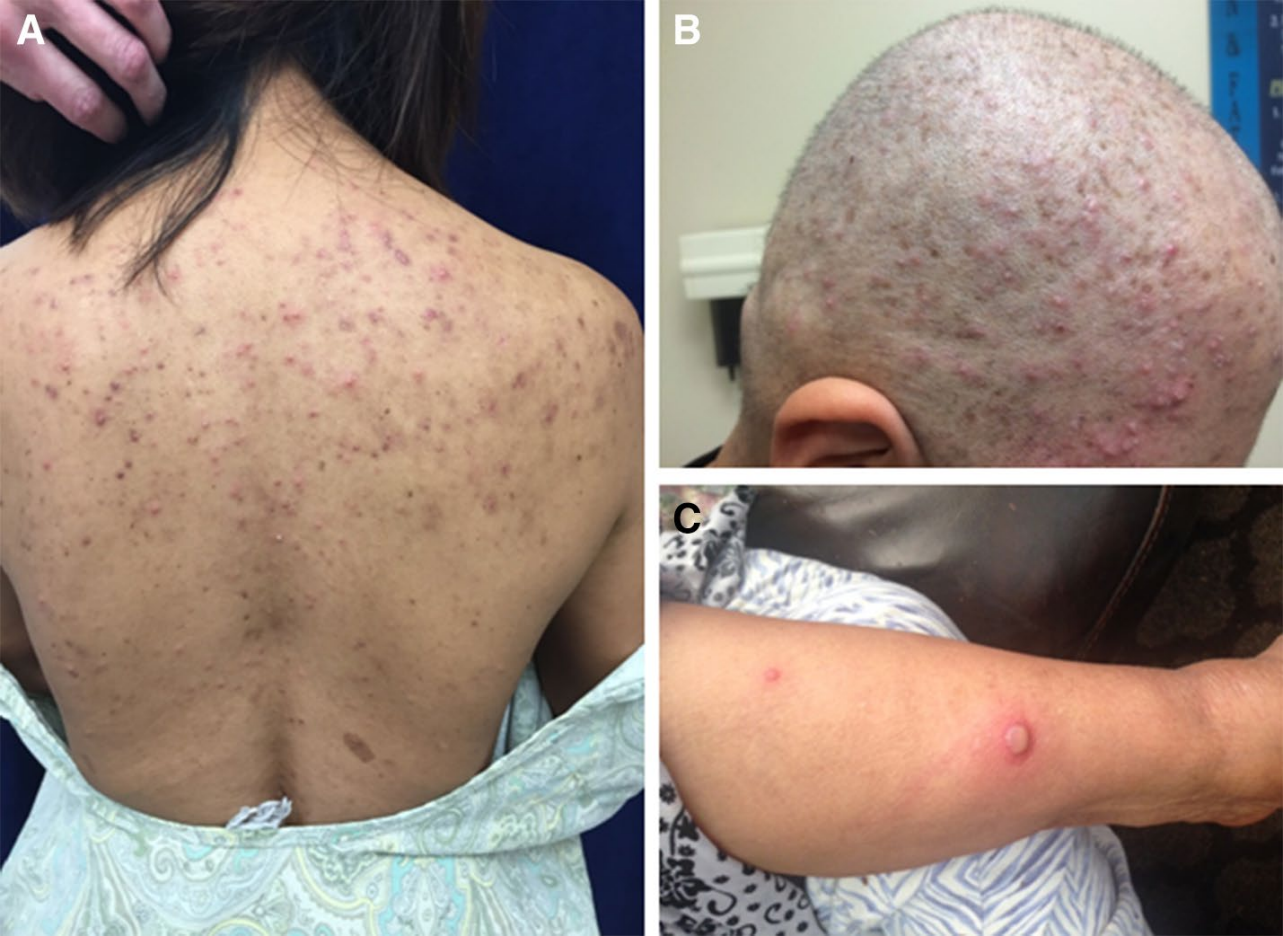
Folliculitis: **a** trunk and back; **b** scalp; **c** large pustule on arm

The most common infection was folliculitis, which developed in 25 (52%) patients within 2–7 days of treatment. The areas of involvement included scalp (13), face (9; 2 also had in chest), abdomen (2), and arm (1). The skin eruptions were self-limited and frequently recurred with each additional cycle. Since our initial report, we have treated infections like EGFR drug reactions using topical clindamycin or doxycycline vs oral or parenteral antibiotics for more severe infections. Examples of the folliculitis are shown in Fig. 1.

### Deep-seated infections

Abscesses developed at different sites in 24 patients: buttocks (4) and one each involving the breast, skin, axilla, vagina, chest wall, thigh, thumb, and shoulder. Two patients developed postoperative wound infections requiring additional surgery. Two patients developed portacath infections within 2 days of catheter placement and required removal of the device. One patient developed a dental abscess and had recurrent episodes of *Streptococcus agalactiae* bacteremia. Her case is discussed in detail below. Most abscesses were treated successfully with antibiotics. One of the patients who had an infected portacath developed tenderness in a prosthetic knee. She was initially treated conservatively with intravenous vancomycin but eventually required removal and replacement of the prosthesis. Cultures preoperatively were negative but methicillin-sensitive *Staphylococcus aureus* was cultured at surgery.

Severe paronychial infections were observed in 7 (15%) patients and required surgical intervention in 1. Cellulitis was observed in 5 (10%) patients and was treated with conservatively with antibiotics.

Most patients were treated for presumed *Staphylococcus* infections without obtaining cultures. Of the 21 infectious episodes for which cultures were obtained, all had infection with Gram-positive bacteria. Methicillin-sensitive *Staphylococcus aureus* (MSSA) was identified in seven patients, methicillin-resistant *Staphylococcus aureus* (MRSA) in eight, *Streptococcus agalactiae* in three (same patient), and *Enterobacter faecalis* in one (toe infection). The cultures were negative in two patients who had already been initiated on antibiotics.

### Fatality

A 58-year-old woman with a locally advanced estrogen receptor positive (ER +), progesterone receptor positive (PR +), HER2-positive cancer received her first cycle of pertuzumab, trastuzumab, carboplatin, and docetaxel (PTCH) without incidence. She was seen on day 1 of cycle 2 with a WBC of 13.0 (11.7 ANC) and received Neulasta^®^ on day2. On day 6, her family reported a sudden change in mental status and she arrived at the local ED in full arrest and could not be resuscitated. No blood was drawn to assess the WBC or obtain cultures. At autopsy, the cause of death was ascribed to sepsis from Gram-positive cocci, which was not speciated.

### Hypogammaglobulinemia

Because of the high incidence of Gram-positive cocci infections, documented or suspected, we questioned whether patients were hypogammaglobulinemic and began measuring quantitative immunoglobulins. Because this was a registry trial, there was no requirement for assessing immunoglobulins and they were measured at different time periods throughout chemotherapy. Of the 33 patients tested for immunoglobulin levels, 14 (36%) had abnormally low levels; IgG was low in 6; IgM in 3, and IgA in 2. Both IgG and IgM were low in 1 and all three immunoglobulins were low in 1.

**Fig. 2:**
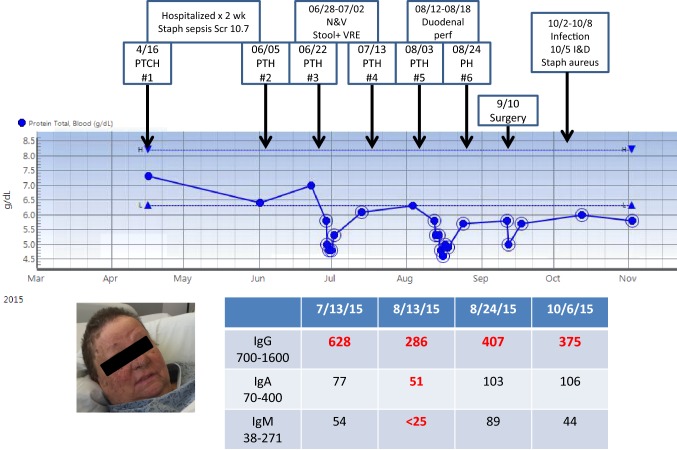
A 52-year-old woman who developed sepsis following cycle 1 of chemotherapy. Treatment cycles and quantitative immunoglobulins and total protein level are charted over time

The following two patients illustrate the impact of pertuzumab on protein levels and infection. The course of a 52-year-old woman (Patient 1) who was treated for locally advanced breast cancer is summarized in Fig. 2, charting her total protein and immunoglobulin levels. On day 14 following cycle 1 of PTCH, she was emergently admitted to the hospital with a facial rash with pustules, loss of consciousness, hypotension, and decreased urine output. On admission, her WBC was 25.3 and she had a Scr of 16.63. She was treated with vasopressors and ceftriaxone with improvement in hemodynamics and renal function without the need for dialysis. She was discharged on day 11 with normal renal function. Subsequent chemotherapy excluded carboplatin. Following cycle 3, she was hospitalized with nausea and vomiting, cultures were negative with the exception of vancomycin-resistant *Enterococcus* (VRE) in her stool that was consistent with colonization. IgG on that admission was low. Cycle 5 was complicated by a duodenal perforation but no documented infection. IgG, IgM, and IgA were low on that admission (what were the levels). Her final preoperative treatment included only pertuzumab and trastuzumab. The postoperative course was complicated by a wound infection requiring incision and debridement with microbiology positive for *Staphylococcus aureus*. Pertuzumab was deleted from her postoperative treatment and immunoglobulins returned to the normal level. She is alive and well and free of disease 39 months from diagnosis and with no further infectious or renal sequelae.

**Fig. 3:**
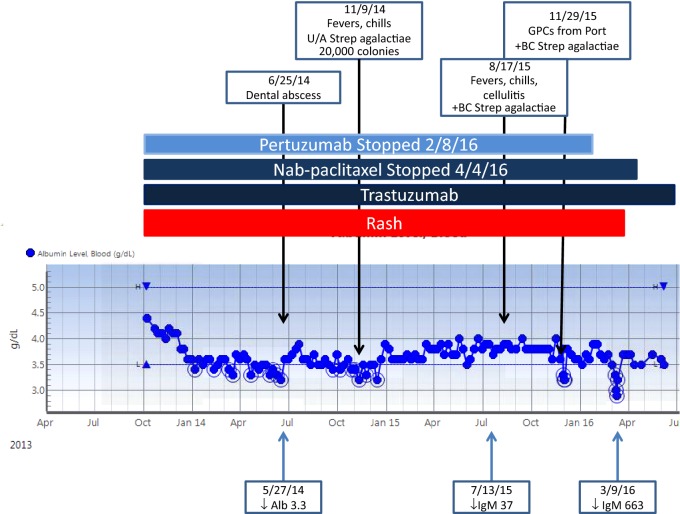
A 31-year-old woman who developed a rash immediately after starting treatment and continued for over 2 years until the pertuzumab was discontinued. She developed a dental abscess and documented Strep Agalactiae sepsis with low albumin and low immunoglobulins which resolved with discontinuation of pertuzumab. Clinical events and immunoglobulin levels are plotted across time and albumin levels

The second patient (Patient 2) presented with de novo stage IV disease treated with nab-paclitaxel, pertuzumab, and trastuzumab. Her clinical course and serum albumin levels are shown in Fig. 3. She developed an erythematous rash over her chest with the first cycle of treatment, which waxed and waned. After month 4, she was treated for a dental abscess. Over an 18-month period, she presented on three separate occasions with fever and chills and was documented to have *Streptococcus agalactiae* bacteremia each time and endocarditis was ruled out. The rash resolved within 2 months of stopping the pertuzumab and she has had no further infections. She is currently on maintenance trastuzumab in continued complete remission 58 months from initial diagnosis.

### Impact of infections on response to therapy

Twenty-four women were treated in the neoadjuvant setting. One patient was lost to follow-up and one died prior to surgery; 23 were evaluable for treatment efficacy. The overall pathologic complete response (pCR) at definitive surgery was 68% (15 of 22 patients), including 10/16 HR + and 5/6 HR − patients.

## Discussion

This updated series provides additional support for an association of Gram-positive bacterial infections and cutaneous manifestations with the addition of pertuzumab to trastuzumab alone or trastuzumab-based chemotherapy. Although the skin reaction is reminiscent of that observed with EGFR inhibitors, the deep-seated infections reported in this series suggest a more serious clinical problem that we believe is related to the development of hypogammaglobulinemia and perhaps abrogation of a specific immune pathway that requires further study.

Because this was a registry trial, we relied on the treating physicians to report infectious complications that were attributable to their therapy. We are not able to determine the incidence of these adverse reactions. Previously, we estimated that skin and nail infections occurred in 30% of patients receiving pertuzumab and trastuzumab therapy and this updated case series supports that number and begs the question why this is not a recognized side effect of this therapy [1] We speculate that the skin “rash” has often been attributed to other causes such as dexamethasone or chemotherapy. Folliculitis was the most common problem and was often described as a “rash”. Table 2 summarizes the incidence of rash reported in randomized clinical trials that tested the addition of pertuzumab to trastuzumab-based treatment, and consistently shows an increased incidence of rash in the pertuzumab arm. The CLEOPATRA trial, first reported in 2012, identified a higher incidence of rash in women assigned pertuzumab, 33.7% compared to 24.2% in those not receiving pertuzumab [2]. A more recent analysis of the CLEOPATRA included an assessment of adverse events after completion of docetaxel. The incidence of rash was higher in the pertuzumab arm, 18.3% compared to 8% [3]. Rashes are reported in a higher incidence with pertuzumab regardless of whether the comparator arm includes chemotherapy, endocrine therapy, or trastuzumab without chemotherapy.

**Table 2:** Incidence of rash in randomized trials in HER2 + breast cancer where the addition of pertuzumab was tested

	Population	Incidence of rash by treatment			
Baselga, 2012 [2] Med f/up 19.3 months	First-line metastasis	Docetaxel, trastuzumab	96/397 (24.2%)	Docetaxel, trastuzumab + pertuzumab	137/407 (33.7%)
Swain, 2015 [3] After docetaxel dc’ed med f/up 50 months	First-line metastasis	Control	21/261 (8%)	Trastuzumab + pertuzumab	56/306 (18.3%)
Urruticoechea. 2016 [11]	Advanced disease after tras–chemo	Capecitabine + trastuzumab	11/218 (5%)	Capecitabine, trastuzumab + pertuzumab	34/228 (14.9%)
Rimawi, 2018 [12]	First-line metastasis	AI + trastuzumab	11/124 (8.9%)	AI + trastuzumab + pertuzumab	22/127 (17.3%)
Gianni, 2016 [13]	Neoadjuvant	Docetaxel, trastuzumab	26/107 (24%)	Docetaxel, trastuzumab + pertuzumab	30/107 (28%)
		Docetaxel, pertuzumab	30/94 (32%)	Trastuzumab + pertuzumab	22/108 (20%)

*AI* aromatase inhibitor

A meta-analysis of 13 randomized trials of trastuzumab in over 10,000 women found a significantly higher risk of high-grade infections and febrile neutropenia in patients receiving trastuzumab compared to those who did not [4]. We hypothesize that the hypogammaglobulinemia (or an undefined pathway) predisposes to the development of infections with Gram-positive bacteria. In our experience, skin erythema with pustules, infection, and hypogammaglobulinemia occur within the first 1–4 days after administration of chemotherapy and generally resolve prior to starting the next cycle of therapy. The early development of hypogammaglobulinemia could explain early onset of pustular rashes and abscesses. In the absence of serious complications, patients are not generally seen in the early post-chemotherapy period and this could also explain why others have not identified the hypogammaglobulinemia. Patient 1 was repeatedly seen shortly after chemotherapy and we were able to document low immunoglobulins on multiple occasions even without a documented infection.

Currently the FDA’s “accelerated approval” process can lead to more rapid drug approval if the preliminary studies demonstrate improvement in surrogate markers of the disease, such as tumor shrinkage. Confirmatory data that address benefit to the patient are required for full FDA approval. Randomized clinical trials are designed to demonstrate the improved efficacy of a therapeutic agent and are generally not powered to identify uncommon side effects [5]. Generally toxicity is assessed during the treatment period and long-term complications are more difficult to identify. Even serious adverse events have been underestimated and many do not become apparent for decades after FDA approval [6, 7]. It should not be surprising that adverse events, even serious adverse events, are often identified years after a drug has been on the market.

Rashes are often reported in patients treated with tyrosine kinase inhibitors (TKIs) in for a variety of cancers. Often the rash has been associated with more favorable disease outcomes and this includes lapatinib in HER2 positive breast cancer [8–10]. Our series was too small to determine whether infectious complications had any prognostic significance. Following neoadjuvant therapy, pCR at surgery was seen in 68% of patients and is comparable to the reported data.

We routinely discuss the risk of infectious complications as part of the chemotherapy teaching for women receiving regimens, which include both trastuzumab and pertuzumab. Patients who develop folliculitis are initially treated with topical clindamycin gel or oral doxycycline in more severe cases and chemotherapy can continue on schedule. It is important that infectious complications be recognized as a side effect of pertuzumab and trastuzumab, because none of our patients were neutropenic, most received white blood cell growth factors, and one of our patients died as a result of a Gram-positive bacterial infection on day 6 of her second cycle of treatment.

The data presented are from a single institution and the mechanism for this complication has yet to be elucidated. We are initiating a clinical trial to assess serial immunoglobulins and granulocyte function after treatment with regimens that include trastuzumab and pertuzumab.
